# A Novel Sleep Staging Network Based on Data Adaptation and Multimodal Fusion

**DOI:** 10.3389/fnhum.2021.727139

**Published:** 2021-10-08

**Authors:** Lijuan Duan, Mengying Li, Changming Wang, Yuanhua Qiao, Zeyu Wang, Sha Sha, Mingai Li

**Affiliations:** ^1^Faculty of Information Technology, Beijing University of Technology, Beijing, China; ^2^Beijing Key Laboratory of Trusted Computing, Beijing, China; ^3^National Engineering Laboratory for Critical Technologies of Information Security Classified Protection, Beijing, China; ^4^Brain-Inspired Intelligence and Clinical Translational Research Center, Beijing, China; ^5^Department of Neurosurgery, Xuanwu Hospital, Capital Medical University, Beijing, China; ^6^College of Applied Sciences, Beijing University of Technology, Beijing, China; ^7^Beijing Anding Hospital, Capital Medical University, Beijing, China; ^8^Faculty of Information Technology, Beijing University of Technology, Beijing, China

**Keywords:** deep learning, HHT, sleep stage classification, multimodal physiological signals, fusion networks

## Abstract

Sleep staging is one of the important methods to diagnosis and treatment of sleep diseases. However, it is laborious and time-consuming, therefore, computer assisted sleep staging is necessary. Most of the existing sleep staging researches using hand-engineered features rely on prior knowledges of sleep analysis, and usually single channel electroencephalogram (EEG) is used for sleep staging task. Prior knowledge is not always available, and single channel EEG signal cannot fully represent the patient’s sleeping physiological states. To tackle the above two problems, we propose an automatic sleep staging network model based on data adaptation and multimodal feature fusion using EEG and electrooculogram (EOG) signals. 3D-CNN is used to extract the time-frequency features of EEG at different time scales, and LSTM is used to learn the frequency evolution of EOG. The nonlinear relationship between the High-layer features of EEG and EOG is fitted by deep probabilistic network. Experiments on SLEEP-EDF and a private dataset show that the proposed model achieves state-of-the-art performance. Moreover, the prediction result is in accordance with that from the expert diagnosis.

## Introduction

SLEEP is an important physiological requirement of human beings, which is essential for human health. Sufficient high-quality sleep guarantees the efficiency of people’s work and study, also it helps people to maintain mental health and revival of physical strength. Conversely, sleep disorders such as sleep deprivation (Bixler, 2009), sleep disordered breathing (Bédard et al., 1991; Aloia et al., 2004) and other related sleep disease (Ivanenko and Gururaj, 2009), including aggression or impulsive behavior, executive dysfunction, memory and attention problems, anxiety and depression, etc., are all associated with neuropsychological deficits (Redline et al., 1997; El-Ad and Lavie, 2005). Nowadays, sleep diseases have become one of the main factors endangering human health. Therefore, sleep detection, analysis and evaluation research has been paid intensive attention in the field of healthcare. The sleep stage classification is one of the key steps to effectively analyze the structure of sleep, diagnose and treat sleep-related diseases (Memar and Faradji, 2018). For example, the division of sleep stages is the first step to diagnose patients with sleep disorders. By observing the changes and patterns of physiological signals during individual sleep, doctors divide adult sleep into different states according to the patterns. A set of sensors are connected to different parts of the patient’s body to collect sleep data and record information such as sleep patterns, breathing, heart activity, and limb movements during sleep. The acquired data is called polysomnography (PSG), and it consists of the patient’s electroencephalogram (EEG), electrocardiogram (ECG), electrooculogram (EOG), electromyogram (EMG) and other physiological signals (Supratak et al., 2017). Generally, by observing the patient’s whole-night PSG sleep recording, sleep experts divide the sleep signal segmented into 30-s epochs into a sleep stage. It is no doubt that it is a huge and laborious job. Therefore, computer-aided sleep stage classification schemes are essential for the diagnosis of sleep-related diseases and sleep monitoring.

As people pay more and more attention to sleep problems, researchers have carried out a lot of studies on sleep staging. In 1924, Hans Berger recorded EEG activity from human brain and scalp for the first time and named it electroencephalogram (Grasser et al., 2011). In 1953, Nathaniel Kleitman and Aserinsky discovered the characteristics of eye movement during sleep, divide them to rapid eye movement (REM) sleep and non-rapid eye movement (NREM) sleep (Jean-Baptiste et al., 2014). In 1968, Allan Rechtschaffen and Anthony Kales divided NREM into four stages using the known R&K rules (Rodenbeck et al., 2010). In 2007, American Academy of Sleep Medicine reformulated a new classification manual for sleep classification called AASM rules, combining the NREM sleep stage 3 (N3) and NREM sleep stage 4 (N4) in the R&K standard (Choi et al., 2010).

The traditional sleep stage classification methods extract features manually from the physiological signals, and then distinguish the sleep stages according to the extracted features. Many hand-made features are designed based on sleep experts’ professional knowledges, including time domain features (Karlen et al., 2009), frequency domain features (Koley and Dey, 2012; Zhao et al., 2019), and time-frequency features (Chouvarda et al., 2011; Hassan and Bhuiyan, 2016; Yucelbas et al., 2018). Peker et al. (2015) studied 41 features that have a significant effect on sleep stages recognition. Anderson et al. (1998) used the autoregressive model to extract EEG signal features, and then used shallow forward neural networks to perform a 10-fold cross-validation experiment on the subjects. The experiment reached an average classification accuracy of 38–71%. Sharma et al. (2017) proposed a sleep staging method based on iterative filtering, and the average accuracy finally reached 86.20%. In terms of classification methods, many traditional classifiers are used in EEG signal detection research and recognition techniques, such as Support Vector Machine (SVM), Linear Discriminant Analysis (LDA) (Subasi and Gursoy, 2010), K-Nearest Neighbor (KNN) (Yazdani et al., 2009), etc. In addition, algorithms such as Independent Component Analysis (ICA) and Principal Component Analysis (PCA) are usually used to improve classification accuracy.

Recently deep learning network are used widely in sleep staging, and the biggest difference between deep learning and traditional pattern recognition methods is that the features are automatically learned from big data, rather than based on manual design. Tsinalis et al. (2016) used CNN to automatically distinguish sleep stages based on single-channel EEG data without using prior knowledge, and the overall accuracy is 74%. Supratak et al. (2017) used CNN to extract the characteristics of EEG, and then they used bidirectional LSTM to learn the transition laws of each stage of sleep, and the overall accuracy is 82%. In another study conducted by Phan et al. (2019) a hierarchical SeqSleepNet was proposed, which took sequences of multiple periods as input and classified them at the same time. Humayun et al. (2019) used a 34-layer deep residual CNN for classification tasks and obtained higher accuracy.

In recent years, Multimodal Fusion has been widely introduced in many fields such as computer vision and Emotion Recognition. In order to comprehensively reflect the sleeping situation, Estrada et al. analyzed the potential characteristics of EOG and EMG at different stages during sleep and verified that EOG and EMG are two important indicators of sleep staging (Estrada et al., 2006). Shimada et al. introduced EEG, EOG, and EMG as inputs into the neural network, and they used the sleep stage features for automatic classification (Boulanger, 1988). Chambon et al. (2018) proposed an end-to-end deep learning approach to perform temporal sleep stage classification using multivariate time series from multiple modalities (EEG, EOG, and EMG). Their proposed approach is particularly good at detecting W (high sensitivity 0.85 and specificity close to 1).

We present a deep learning fusion network framework denoted as Multi Sensor Deep Fusion Network (MSDFN) for feature extraction, multimodal fusion and sleep stage classification. The main contribution can be summarized as follows: On the one hand, the research eliminates the dependence on prior knowledge in feature extraction stage by introducing efficient adaptive signal analysis; On the other hand, the work reflects the differences between sleep stages comprehensively by using multimodal sleep data. In order to improve the traditional fusion methods such as sum and splicing, we use depth probability model to fit the high-order nonlinear correlation between different modal features, and integrates the representation of multimodal sleep data.

The rest of the paper is organized as follows: in section “Sleep Stage Classification Model,” we introduce the proposed model of sleep staging. Section “Dataset and Assessment” depicts the datasets and evaluation indicators used in this paper. In Section “Experiment and Analysis,” the related experiments of the proposed method are described. Finally, in section “Discussion and Conclusion,” the discussion and conclusions are given.

## Sleep Stage Classification Model

**FIGURE 1:**
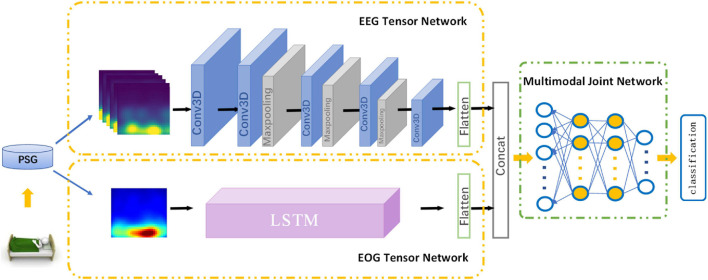
Architecture of MSDFN.

In this section, we introduce MSDFN in detail. To collect more discriminative information, Hilbert–Huang transform is used to extract the time-frequency characteristics of the original signal. To learn heterogeneous features, two parts of the architecture are designed for EEG and EOG signal, respectively. Then the Multimodal Fusion Networks are established to explore the intermodal interactions between multimodal data. The structure of proposed method is shown in Figure 1.

### Hilbert–Huang Transform

Hilbert–Huang transform (HHT) is an effective and adaptive time-frequency analysis method, which is suitable for nonlinear and nonstationary time series. The signal is decomposed to components denoted as Intrinsic Mode Functions (IMFs) through using the algorithm proposed by Huang (Huang et al., 1998). After that, we can utilize the Hilbert transform (HT) for each IMF to obtain the distribution of the amplitude or energy in time-frequency field.

The EMD algorithm directly decomposes the original signal according to the characteristics of the signal, thereby adaptively decomposing the signal into several IMF components. It is different from Fourier transform and wavelet-based transform as HHT has the feature of locally defining nonstationary data and has become a more popular method in recent years. In this study, both EEG and EOG perform time-frequency transformation. The difference is that the 30 s EEG signal will be divided into 5 s sub-segments to extract six time-frequency information matrices as an EEG sample, but the 30 s EOG is transformed as a whole.

### Heterogeneous Feature Learning Network

The part of Heterogeneous feature learning of MSDFN contains EEG Tensor Network (ETN) and EOG Tensor Network (OTN).

Electroencephalogram Tensor Network takes HHT time-frequency features based on EEG signals as input and generates the corresponding EEG signal tensor. It is derived from 3DCNN, which is widely used in the field of video classification and action recognition. In this paper, the 30 s original EEG signal is further divided into six sub-segments of 5 s, which are converted into image-like data through the above-mentioned time-frequency feature processing and stacked as input for 3D CNN, to extract the deep representation while respecting the locality within the feature map in three dimensions. The sub-network contains four convolutional layers and three pooling layers. The convolutional layer is used to learn high-level representation of EEG features. It contains three sizes of 3D convolution kernels, the size is 2 × 5 × 5, 2 × 3 × 3, 2 × 1 × 1, and Conv3D_1, Conv3D_4, Conv3D_5 are used to increase the number of feature maps. All layers extract a more abstracted representation of the feature.

Electrooculogram Tensor Network is used to process the EOG signal data, taking the time-frequency characteristics generated by the 30 s EOG signal as input. Setting the input size to 30, timesteps to 30, the number of hidden units in the network to 200, and cyclically entering the values that change with time at different frequencies. While evaluating sleep stages, sleep experts refer to the data of other modalities at the same time to obtain a more accurate assessment. We use the OTN output based on LSTM to characterize the progressive relationship of the EOG, and use the output as a heterogeneous feature to fuse with the output of the ETN.

Sleep data has sample imbalance, because the length of time in each stage is different during sleep. In order to solve this problem, Focal Loss is used for the above network to reduce the proportion imbalance between sample categories. And the loss function is given as follows:


(1) $$L\,o\,s\,s_{f\,l}=-\alpha_{l\,a\,b\,e\,l}\,{\left(1-p_{p\,r\,e}*p_{l\,a\,b\,e\,l}\right)}^{\gamma }\,\log\left(p_{p\,r\,e}\right)$$

where α_*label*_ is a balance factor, which is used as an equilibrium between samples of different classes, and γ is used to adjust the rate of weight reduction of simple samples, which solves the problems of simple samples and difficult samples.

### Multimodal Fusion Network

Many deep learning networks based on shallow fusion or decision fusion are applied to multimodal fusion technology. However, these methods cannot effectively simulate the complex nonlinear joint distribution and are difficult to capture the intrinsic correlation among different modalities, because these modes are considered independent. To construct a network that learns complex joint feature representations, we use multimodal fusion network based on Deep belief network (DBN) to learn the nonlinear relationship between EEG and EOG.

**FIGURE 2:**
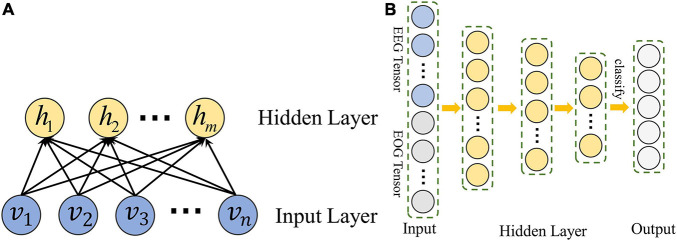
The structure of DBN constructed in MSDFN. **(A)** The structure of RBM; **(B)** The structure of DBN.

A set of restricted Boltzmann machines (RBMS) is the main component of DBN. The structure of RBM is shown in Figure 2. It contains input units *v* ∈ {*v_i_}^R^* and hidden units *h* ∈ {*h_j_}^R^*.

With a given set of values (*v*,*h*), we can define the model with an energy function as follow:


(2) $$E\,\left(v,h\right)=-\sum_{i\in i\,n\,p\,u\,t}\frac{{\left(v_{i}-a_{i}\right)}^{2}}{2\,\sigma_{i}^{2}}\,v_{i}-\sum_{j\in h\,i\,d\,d\,e\,n}b_{j}\,h_{j}-\sum_{i,j}\frac{v_{i}}{\sigma_{i}}\,h_{j}\,w_{i\,j}$$

where *v*_*i*_ and *h*_*j*_are the value of input unit *i* and hidden unit *j*;*a*_*i*_ and *b*_*j*_are the biases of *v*_*i*_ and *h*_*j*_; and *w*_*ij*_ is the weight between *v*_*i*_ and *h*_*j*_;σ_*i*_ is the standard deviation of Gaussian noise for a real valued input unit. We can calculate the conditional distribution:


(3) $$p\left(v_{i}=1|h\right)=N\left(b_{i}+\sigma_{i}\sum_{j}W_{i\,j}h_{j},\sigma_{i}^{2}\right)$$

(4) $$p\left(h_{j}=1|v\right)=N\left(b_{j}+\sum_{i}W_{i\,j}v_{i}\right)$$

Hidden layer vectors of each RBM in DBN are used to train another RBM, thus we can use DBN to capture the high-order nonlinear correlation of the input data. Finally, back propagation is adopted to train the entire DBN with Cross Entropy loss function:


(5) $$L\,o\,s\,s_{c\,e}=-\sum_{i=1}^{k}y_{i}\,\log\left(p_{i}\right)$$

where *k* is the number of classes, *y*_*i*_ and *p*_*i*_ are the label and probability of class *i*.

## Dataset and Assessment

### Datasets

#### SLEEP-EDF

The multimodal PSG dataset used in this research comes from the Sleep-EDF Expanded databases (Kemp et al., 2000) provided by PhysioNet (Goldberger et al., 2000). The Sleep-EDF Expanded database contains 197 whole-night PolySomnoGraphic sleep recordings, including EEG signals with electrodes located on Fpz-Cz and Pz-Oz, respectively, horizontal EOG signals, other sleep physiological signals, and manually labeled events. In most cases, the PSGs of each subject is recorded for approximately 20 h in two nights. The EOG and EEG signals were each sampled at the frequency of 100 Hz, and manually annotated by experts into different stages, which are converted to five stages according to the AASM standard in this work. In order to avoid the influence of other additional factors on this research, this paper only uses the data of 20 subjects obtained by monitoring of the healthy people.

#### Private Data

The experiment was approved by the Ethics Committee of Xuanwu Hospital, Capital Medical University. All the participants signed consent forms for participation and were fully informed of the experimental and data acquisition procedures. This experiment uses the multimodal PSG data provided by Xuanwu Hospital, Capital Medical University. This data set includes whole-night PSG recordings of 20 subjects. By using Compumedics Grael, PSG of each subject was collected about 10 h of sleep recording for one night. The electrode placement method recommended by AASM for data collection includes seven EEG channels, two EOG channels, EMG, ECG and other sleep signals. Among them, EEG and EOG use a sampling frequency of 1,000 Hz, and the data is also artificially marked as five sleep stages.

### Data Preprocessing

According to the AASM sleep staging rules, well-trained professional sleep physicians classify the patients’ sleep stages by the following steps:

(1) The PSG data of the whole night is divided into 30 s PSG segments without overlap, which is used consistently with the sleep staging process following the AASM guidelines;
(2) Sleep physicians use professional software to visually display these 30 s PSG segments, then determining the sleep stages according to relevant criteria by observing these segments.

In this study, the sleep data of each 30 s is also used as an independent sleep staging sample. For the purpose of preprocessing of raw data, EEG and EOG signals are filtered with 3th order butter-worth bandpass filter with the cut-off frequencies of 0.5–32 Hz and 0.5–10 Hz, respectively. In addition, the sampling frequency of private data is 100 Hz after down-sampling, and the bad segments caused by electrode falling off are removed. Finally, we extract 49,853 samples from the SLEEP-EDF data and 22,014 samples from the private sleep data. Each sample is indicated by its own sleep staging label. Table 1 shows the information of the constructed sample sets. From Table 1, it can be seen that the proportion of each sleep stage in two data sets conforms to the sleep structure of healthy people.

**TABLE 1:** The distribution of samples in data sets.

**Sleep stages**	**W**	**N1**	**N2**	**N3**	**REM**	**Total**
SLEEP-EDF	15,257	4,339	19,014	4,009	7,234	49,853
Private data	4,831	1,997	10,440	1,239	3,507	22,014

### Evaluation

In order to evaluate the effectiveness of the proposed method, we calculate confusion matrix to show difference between the results given by the proposed method and the expert’s mark, which are given as


(6) $$C\,M=\left[\begin{array}{ccccc} S_{11} & S_{12} & S_{13} & S_{14} & S_{15}\\ S_{21} & S_{22} & S_{23} & S_{24} & S_{25}\\ S_{31} & S_{32} & S_{33} & S_{34} & S_{35}\\ S_{41} & S_{42} & S_{43} & S_{44} & S_{45}\\ S_{51} & S_{52} & S_{53} & S_{54} & S_{55} \end{array}\right]$$

where *S*_*ij*_ represents the number of fragments marked as sleep stage *i* and classified as sleep stage *j*. The value of *i* or *j*. indicates that the period is equal to 1, 2, 3, 4, and 5, respectively, indicating sleep stages of W, REM, N1, N2, and N3.

Cohen’s Kappa coefficient is also used to measure the consistency between the classification result and the expert’s mark. The calculation formula is as follows:


(7) $$K\,a\,p\,p\,a=\frac{P_{0}-P_{e}}{1-P_{e}}$$

(8) $$P_{0}=\frac{\sum_{i=1}^{5}S_{i\,i}}{\sum_{i=1}^{5}\sum_{j=1}^{5}S_{i\,j}}$$

(9) $$P_{e}=\frac{\sum_{i=1}^{5}\left(\sum_{j=1}^{5}S_{i\,j}\,\sum_{j=1}^{5}S_{j\,i}\right)}{{\left(\sum_{i=1}^{5}\sum_{j=1}^{5}S_{i\,j}\right)}^{2}}$$

In this paper, we will also calculate accuracy (AC) and F1 score (F1) to show the performance of the proposed method as shown in Equation (17) to (18).


(10) $$A\,C=\frac{T\,P+T\,N}{T\,P+T\,N+F\,P+F\,N}$$

(11) $$F\,1=2\cdot \frac{P\cdot R}{P+R}$$

AC is also used to represent the percentage of the correct classification period to all test periods. In this paper, AC and F1 scores are used as indicators to evaluate the classification effect of each sleep stage. The AC and Kappa coefficients are used to evaluate the overall performance of all stages and measure the consistency between the algorithm prediction results and the expert score results.

## Experiment and Analysis

In this section, we introduce a series of comparative experimental results of sleep stage classification. These experiments focus on multi-class classification problems:

(1) Classification experiments based on multi-modal data with single channel;
(2) Classification experiments based on multi-modal data with multi-channels;
(3) Multi-Classification experiments.

The process of the experiment details are given in the following subsections.

**FIGURE 3:**
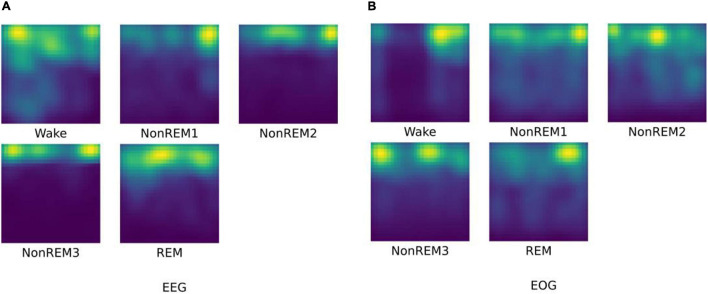
The HHT result for different sleep stages. **(A)** visualization of EEG HHT results; **(B)** visualization of EOG HHT results.

First, we visualize the time-frequency features extracted by HHT, as shown in Figure 3. As a common time-frequency analysis method, the biggest advantage of HHT lies in its strong adaptability, which is suitable for the input of end-to-end feature network learning. It overcomes the shortcomings of traditional time-frequency methods that require manual parameter adjustment, and it can adaptively process non-linear and non-stationary physiological signals with high characteristics.

As we have seen, the time-frequency characteristics of EEG can reflect the differences of various sleep stages. The HHT of W and N1 reflect the low energy value of high-frequency components. For N2 and N3 sleep stages, relatively low-frequency and high-amplitude waves with higher energy appear. In the REM, high-frequency waves appear again, and the signal energy is reduced relatively. For EOG, it can be found that the high-frequency eye movement gradually disappears from W to N2, and rapid eye movement occurs again in the REM period. This information is basically in line with the focus of professional physicians in the sleep scoring process. It is worth noting that the extracted EOG features of N3 reflects continuous low-frequency information, but there is usually no eye movement in N3 phase. Therefore, we speculate that it may be due to the stronger brain activity as of N3 has an impact on the EOG signals during the signal acquisition process.

We show the network parameters of MSDFN in Table 2. Hyper-parameters for 3D convolutional layers are kernel size in 3D, strides in 3D, and the number of filters. For 3D max-pooling layers they are pool sizes and strides. For LSTM they are the length of sequence, the dimensionality of the feature vector at each time step, and the number of hidden units. For fully-connected layers they are numbers of input and hidden units. In addition, we use Focal Loss as the loss function of ETN and OTN. In this function, we set alpha weight to (0.6, 0.9, 0.6, 0.9, and 0.8) for SLEEP-EDF and (0.8, 0.9, 0.5, 0.9, and 0.8) for private data, and gamma to 2. The Adam optimization approach is used to train the sub-network model with learning rate of 1e-3, and categorical cross-entropy is used to train the fusion network. Learning rate in fusion networks is set to 1e-3. Finally, softmax activation is used for the output layer. L2 regularization is applied to weights of all convolutional and fully-connected layers. All weights are initialized randomly from He Normal initializer (He et al., 2015) which is more suitable for ReLU activation. Batch normalization is performed after every convolutional layer and before their activation. The size of minibatch is 128 and the number of epochs is set to 50.

**TABLE 2:** The parameters of proposed model.

**Layer**	**Hyper- parameters**	**Activation function**	**Output shape**
Conv3D 1	(2, 5, 5), (1, 2, 2), 16	ReLU	6 × 15 × 15, 16
Conv3D 2	(2, 5, 5), (1, 2, 2), 16	ReLU	6 × 8 × 8, 16
Max-pooling 1	(1, 2, 2), (1, 2, 2)	–	6 × 4 × 4, 16
Conv3D 3	(2, 3, 3), (1, 2, 2), 16	ReLU	6 × 2 × 2, 16
Max-pooling2	(1, 2, 2), (1, 1, 1)	–	6 × 2 × 2, 16
Conv3D 4	(2, 3, 3), (1, 2, 2), 32	ReLU	6 × 1 × 1, 32
Max-pooling 3	(2, 1, 1), (1, 1, 1)	–	6 × 1 × 1, 32
Conv3D 5	(2, 1, 1), (1, 1, 1), 64	ReLU	6 × 1 × 1, 64
LSTM	[(30, 30), 200]	–	200
DBN hidden 1	(584, 300)	Sigmoid	300
DBN hidden 2	(300, 200)	Sigmoid	200
DBN hidden 3	(200, 100)	Sigmoid	100

### Classification Experiments Based on Multimodal Data of Single-Channel

In this subsection, the influence of a single modal network on the classification of combined sleep stages is given. In our work, 15 subjects in the SLEEP-EDF dataset are adopted, and 10-fold cross-validation of the leave-one-out rule is used to verify performance. We carry out the experiment research following the AASM rule which sets the number of categories to 5. Each subject is independent for the testing, and the remaining 14 subjects are merged into a training set. During this process, the test results of 10 subjects are randomly selected for the evaluation. We also compare the results of the proposed method with the latest results on the same data set.

**FIGURE 4:**
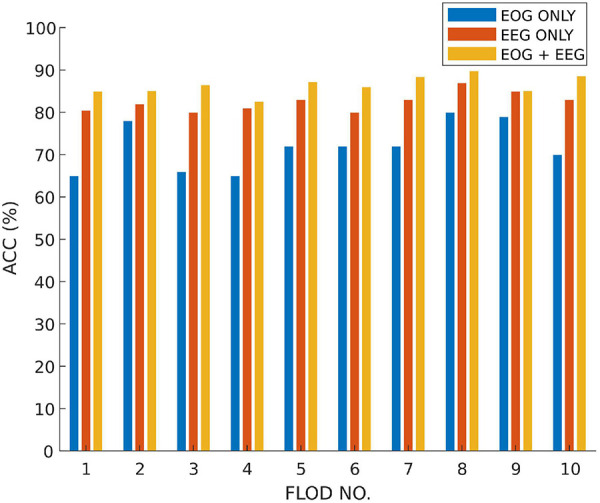
The accuracy of classification experiments based on SLEEP-EDF.

In order to verify the fusion effect of the proposed model, we compared the results of single-modal data classification and multi-modal data fusion classification. The results are shown in Figure 4. We use ETN to classify EEG signals and OTN to classify EOG, respectively. Obviously, the classification result of MSDFN is significantly better than the result of the separate classification of the two modalities. Among them, the average accuracy of the fusion method is 4.03% higher than that of the classification using only ETN, and the average accuracy of the results of classification using only OTN is 14.53% lower.

**FIGURE 5:**
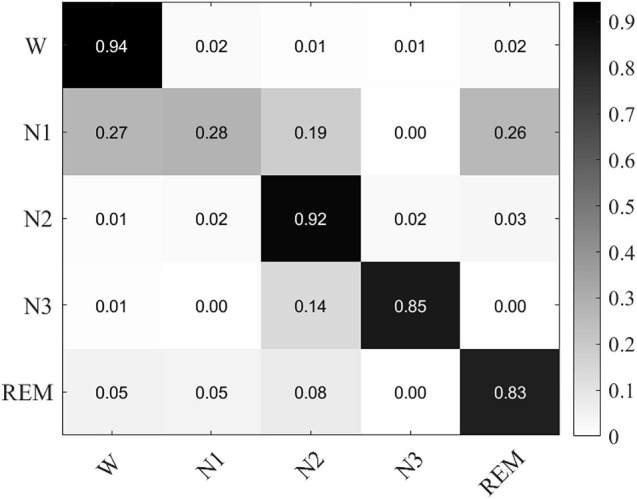
The confusion matrix of classification experiments result based on single channel multimodal data.

The confusion matrix of MSDFN is drawn to analyze the classification accuracy between different categories. The result is shown in Figure 5. Our model achieves good classification results in the four categories of W, N2, N3, and REM. Among them, the category with the best classification performance is W, with an accuracy rate of 94.34%, and the category with the worst classification performance is REM, with an accuracy rate of 82.6%. However, the classification accuracy of N1 is 28.9%. It can be seen that the accuracy of N1 is low because the discriminative characteristics of N1 are similar to those features of W and REM. This is also one of the difficult problems to be solved in sleep multi-classification tasks.

Besides, HHT and CWT features are used as inputs of the network to verify the effectiveness of the model. Table 3 shows the results. The results show that our model is more suitable for HHT. It is obviously that the analysis results of wavelet transform are very dependent on the selection of wavelet bases and related parameters. HHT is obtained by frequency domain component decomposition based on the characteristics of data.

**TABLE 3:** The performance of MSDFN using HHT and CWT.

**Input**	**Accuracy (%)**	**Kappa**	**Fl**
HHT	85.9	0.81	0.86
CWT	80.3	0.77	0.79

### Classification Experiments Based on Multimodal Data With Multi-Channel

In this section, multi-modal and multi-channel data are used to verify the effect of the fusion network. Further, we use a private data set to test the robustness of the model. For the experiment, we use dual-channel EEG and horizontal EOG of 15 subjects in the SLEEP-EDF data set. In another private data set, we used 7-channel EEG data located in F3-M2, F4-M1, M1-M2, C3-M2, C4-M1, O1-M2, O2-M1, and dual-channel EOG contains LEOG and REOG. The training set and test set are divided in the same way as before.

**FIGURE 6:**
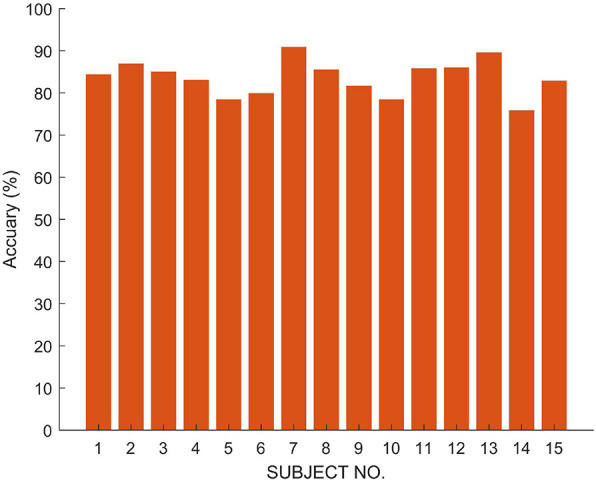
The accuracy of classification experiments based on Private Data.

The results are shown in Figure 6 and Table 4, respectively. The results show that our model has an average accuracy rate of 87.5% on the SLEEP-EDF data set, and an average accuracy rate of 83.8% on the private data set. As it is seen, compared with the experiments based on single channel multi-modal data, the effect of the model using multi-channel data is slightly improved. And the proposed structure has good robustness, and its performance is also stable on real data.

**TABLE 4:** The performance of MSDFN using multi-channel data.

**Data set**	**Accuracy (%)**	**Kappa**	**F1**
SLEEP-EDF	87.5	0.83	0.86
Private data	83.8	0.81	0.83

### Multi-Classification Experiments

**FIGURE 7:**
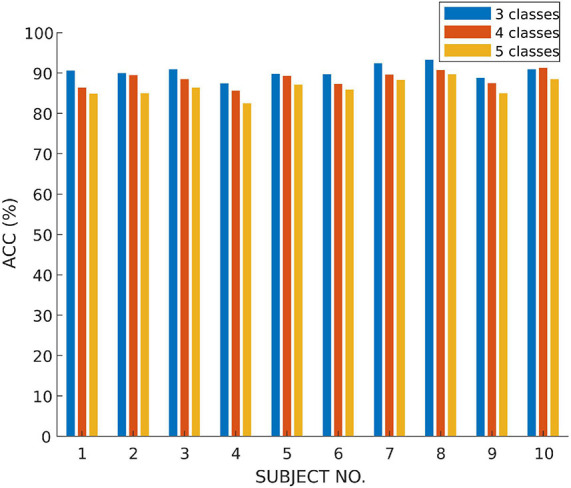
The accuracy of multi-class classification experiments based on SLEEP-EDF.

In this subsection, we use the proposed MSDFN to conduct a comparative experiment with different categories. The results of the categories in this experiment are recorded in the Table 5. Finally, the results can be checked in Figure 7 and Table 6.

**TABLE 5:** The details of categories in multi-classification experiments.

**Class num**	**Categories**
3	W, NREM (N1, N2, and N3) and REM
4	Wake, light sleep (N1, N2), deep sleep (N3), and REM
5	Wake, N1, N2, N3, and REM

**TABLE 6:** The performance of multi-class classification task based on SLEEP-EDF.

**Class num**	**Accuracy (%)**	**Kappa**	**F1**
3	90.5	0.85	0.90
4	88.7	0.84	0.89
5	85.9	0.81	0.86

The performance of the model is verified on tasks with different numbers of categories. It can be seen that with the refinement of the sleep stages, the capability of the model gradually decreases. Among them, the accuracy of three classification is 1.8% higher than that of four classification and 4.6% higher than that of five classification. Therefore, our model has a better classification for most sleep stages, but it does not perform well on distinction for N1 and N2 sleep stages.

**FIGURE 8:**
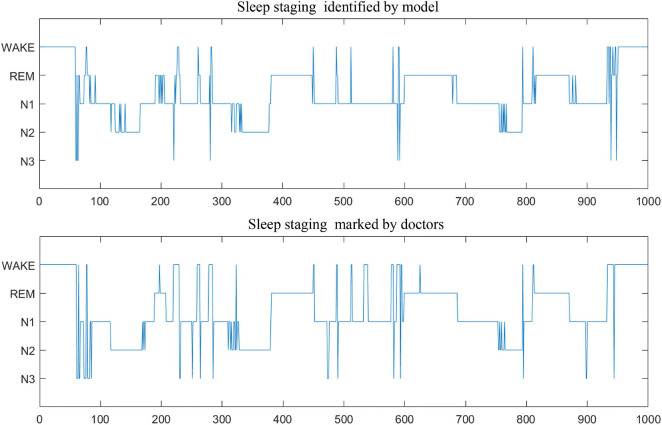
The comparison of sleep stage classification.

In addition, Table 7 compares the effectiveness of some existing sleep stage classification models. In these models, some manual features are extracted for decision to obtain the highest classification results. These manual feature combinations rely heavily on prior knowledge, and it is often difficult to find effective features for end-to-end systems. Our method is based on adaptive time-frequency features and deep fusion network. Compared with the end-to-end deep learning model using single-channel and multi-channel sleep data, it still achieves good classification results. On two different data sets, our model can achieve better overall performance at all stages, and the prediction results are in good agreement with the expert score results. Therefore, MSDFN is a better supplementary model to the existing sleep stages classification model. And Figure 8 shows the comparison between sleep structure analyzed by our model and the label marked by doctors.

**TABLE 7:** Comparison of different methods in classification of sleep stages.

	**Method**	**Input data**	**Feature type**	**Subjects**	**Overall accuracy(%)**
Rodriguez-Sotelo et al., 2014	k-NN	Fpz-Cz + Pz-Oz	Hand-crafted	20 SC	80.0
Andreotti et al., 2018	ResNet	Fpz-Cz + hor. EOG	Learned	20 SC	76.8
Mikkelsen and De Vos, 2018	Deep CNN	Fpz-Cz + hor. EOG	Learned	20 SC	84.0
Phan et al., 2018	Multitask 1-max CNN	Fpz-Cz + hor. EOG	Learned	20 SC	82.3
This work	Deep Fusion framework	Fpz-Cz + hor. EOG	Learned	20SC	**85.9**
This work	Deep Fusion framework	Fpz-Cz + Pz-Oz + hor. EOG	Learned	20SC	**87.5**

*State-of-the-art results are marked in bold.*

## Discussion and Conclusion

This paper proposes an automatic sleep stage classification framework based on HHT and deep multimodal feature fusion network, with the function of data adaptation and multimodal feature fusion. For the work, sleep stages are classified into wakefulness, N1, N2, N3 of non-Rapid Eye Movement (NREM), and REM. By developing a deep learning fusion model based on 3D Convolutional Neural Network (3DCNN), LSTM, and Deep Brief Network (DBN), we accurately and efficiently classify sleep stages with polysomnographic (PSG) data including electroencephalogram (EEG), electrooculogram (EOG). The proposed model is employed to characterize the high-order correlation of the multimodal PSG data, which utilize the Deep Brief Network (DBN) for its strengths of dealing with Non-linear relationship of heterogeneous data. Overall classification accuracy, Cohen’s kappa coefficient and F1 score in SLEEP-EDF are 87.5, 0.83, and 86.3%, respectively. The experimental results show that our method has poor classification result for N1 and REM. The reason is from the similarity of the significant characteristics of the two sleep stages, which is also the challenge we will solve in the next work. In general, MSDFN can achieve good results on both two data sets. The performance of the fusion network compared to the single modal subnetwork has been greatly improved.

## Data Availability Statement

Publicly available datasets were analyzed in this study. This data can be found here: https://www.physionet.org/content/sleep-edfx/1.0.0/.

## Ethics Statement

The studies involving human participants were reviewed and approved by the Ethics Committee of Xuanwu Hospital, Capital Medical University, Beijing, China. Only de-identified data contributed to the analysis. The patients/participants provided their written informed consent to participate in this study. Written informed consent was obtained from the individual(s). The collection of these data meets the biosecurity standards, which is a routine medical examination method without radiation and no additional medication.

## Author Contributions

CW and LD contributed to the conception and design of the study. MYL and ZW designed the network model, carried out the experiment, and wrote the manuscript. YQ and MAL revised the manuscript. SS provided medical background knowledge and interpretation of the results. All authors contributed to the article and approved the submitted version.

## Conflict of Interest

The authors declare that the research was conducted in the absence of any commercial or financial relationships that could be construed as a potential conflict of interest.

## Publisher’s Note

All claims expressed in this article are solely those of the authors and do not necessarily represent those of their affiliated organizations, or those of the publisher, the editors and the reviewers. Any product that may be evaluated in this article, or claim that may be made by its manufacturer, is not guaranteed or endorsed by the publisher.
